# Impact on Quality of Life in Patients who came with Androgenetic Alopecia for Hair Transplantion Surgery in a Clinic

**DOI:** 10.31729/jnma.3500

**Published:** 2018-08-31

**Authors:** Rupak Bishwokarma Ghimire

**Affiliations:** 1 Department of Dermatology, Kathmandu Medical College, Sinamangal, Kathmandu, Nepal

**Keywords:** *androgenetic alopecia*, *hair transplantation surgery*, *quality of life*

## Abstract

**Introduction:**

Androgenetic alopecia, also known as as male pattern baldness, affects up to 50% of men and 10% females worldwide. Patients with baldness seem to have a great impact on quality of life including their self-esteem, confidence, relationship as well as work.

**Methods:**

This is a cross-sectional descriptive study on dermatology quality of life index in patients with androgenetic alopecia who came for hair transplantation surgery at Aavaran Skin Clinic Pvt Ltd, Battisputali, Kathmandu between 15^th^ July 2017 to 15^th^ February 2018. Ethical clearance was taken from ERB of Nepal Health Reasearch Council. All cases enrolled for transplant surgery during the study period and meeting the inclusive criteria were included.

**Results:**

A total of 120 patients participated in the study. Age of the patients ranged from 19 to 49, mean age being 31.87±6.8. Maximum number of patients were in age group 25 to 34. Mean dermatology quality of life index score was 2.79. Maximum score was 14 & minimum score was 0. Maximum effect was seen in question number 2 of self-consciousness, which had impact on 58 (48.33%) patients at some level. Minimum impact on quality of life was seen in sexual activity where only 4 (3.33%) of patients were affected.

**Conclusions:**

Androgenetic alopecia had a small effect on quality of life of our patients, but for some it had a great psychological impact not only with their personal feelings but also with the social response towards their problems.

## INTRODUCTION

Androgenetic alopecia (AGA) also known as male pattern baldness, affects up to 50% of men worldwide.^1^ Medically, alopecia is viewed as a relatively mild condition but those suffering from the condition feel a major distress on life and how other people view them.^2^ Lifestyle and behavioral patterns may contribute to the occurrence and severity of AGA.

In a study done by Alfonso et al. among patients with AGA, majority of them reported that hair loss affected their personal attractiveness and social life.^3^

Williamson et al. reported low self-esteem and loss of self-confidence.^4^ Reid et al. demonstrated that patients rate their hair loss as more severe than dermatologists. Consequently, understanding the psychosocial concern and quality of life (QoL) of patients with AGA has become a matter of great concern.^5^

The objecive of the study was to assess the overall impact in quality of life in patients with AGA using dermatology quality of life index (DLQI).^6^

## METHODS

This study is a cross-sectional descriptive study which was conducted on patients who came for counseling and treatment for hair disorders at Aavaran Skin Clinic Pvt Ltd, Battisputali, Kathmandu between 15^th^ July 2017 to 15^th^ February 2018. Ethical approval was taken from ERB of Nepal Health Reasearch Council. All cases diagnosed as androgenetic alopecia came for hair transplantation surgery, during the study period and meeting the inclusive criteria were included. Patients with diagnoses other than androgenetic apolecia were excluded in the study. The DLQI questionnaire consisted of 10 questions regarding symptoms and feelings, daily activities, leisure, work and school, personal relationships, and treatment as dimensions of life. Each answer was scored on a scale of 0–3 points. Scores were added to yield a total DLQI of 0–30 points; higher scores indicated greater impact on the patient's QoL. The data were processed using an SPSS software package (SPSS 13.0 Inc., Chicago, IL, USA).

## RESULTS

**Figure 1.**
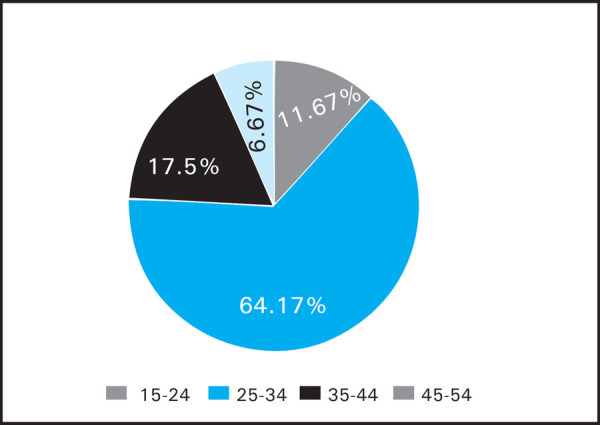
Age Distribution among clients.

A total of 120 patients participated in the study. All patients were males. Mean DLQI score was 2.79. Maximum score was 14 and minimum score was 0. Age of the patients ranged from 19 to 49, mean age being 31.87±6.8. Maximum number of patients was in age group 25 to 34 (Figure 1).

**Figure 2.**
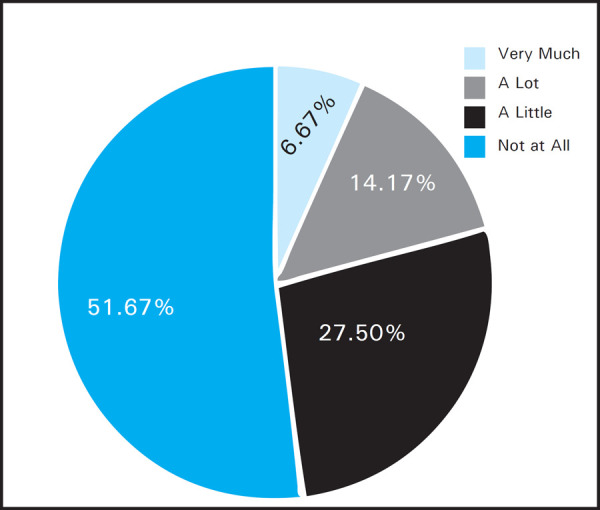
Impact on self-consciousness.

Maximum effect was seen in question number 2 of self consciousness which had a impact on 58 (48.33%) patients at some level, among which where 8 (6.67%) pateints said they were affected very much, 17 (14.17%) were affected a lot and 32 (27%) were affected a little (Figure 2).

**Figure 3.**
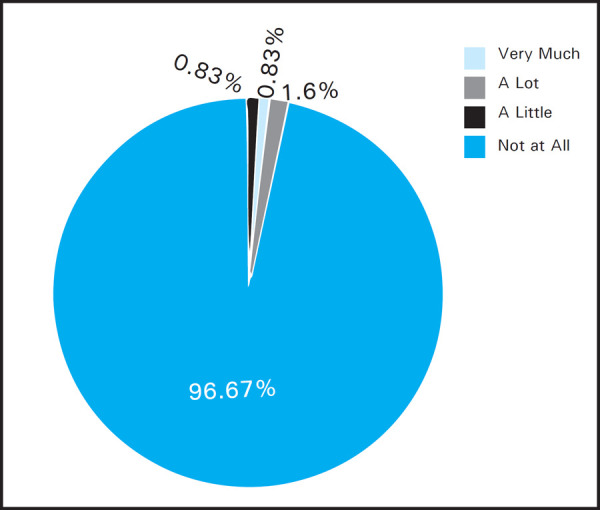
Impact on difficulties with sexual life.

Minimum impact on quality of life was seen in sexual activity where only 4 (3.33%) of patients where affected among which, 1 patient was affected a lot, one very much and two patients said a little affect (Fig. 3).

## DISCUSSION

In our study, the mean score of DLQI was 2.79, and this is taken to represent a small effect on patient's life. In a study conducted by Zhang et al, in China, 178 patients with AA and AGA in this study, the DLQI scores ranged from 0 to 28, with a mean score of 6.3±6.3, which means a moderate effect. Topics in questions 2 (embarrassment), 5 (social or leisure), and 7 (work or study) had the most impact on patients with hair loss, which was similar to our study. The lowest impacts were noted on questions 6 (sports), 8 (relationships), and 1 (symptoms) whereas ours was noted with question 9 related to sexual activity.^7^

Qi et al studied 698 Chinese patients with AA and found that the mean DLQI score was 5.8±5.6.^8^ A study by Williamson et al that included 70 patients with alopecia showed that the mean score of DLQI was 8.3±5.6. They found that the DLQI scores of patients with alopecia were similar to those of patients with severe psoriasis.^4^ Cartwright et al studied 300 patients with severe Alopecia Areata and found that the mean DLQI score was 13.5.^9^ These differences in findings might be due to the differences in the type of alopecia patients selected and in the degrees of severity of these alopecia patients. The present study had several limitations. All the patients in our study were the ones visiting a dermatology clinic seeking hair transplantation surgery, and selection bias may, therefore, have affected the results. Patients were all males, which could be because androgenetic alopecia occurs in less than 10% females and those having the problem may not be ready for hair transplantation. Also, the study sample was relatively small compared to the total population of alopecia patients in Nepal.

## CONCLUSIONS

AGA had a small effect on QoL of our patients, but for some it had a great psychological impact not only with their personal feelings but also with the social response towards their problems.

## Conflict of Interest

**None.**

## References

[1] Thomas J. (2005). Androgenetic Alopecia - Current status. Indian J Dermatol..

[2] Cartwright T, Endean N, Porter A. (2009). Illness perceptions, coping and quality of life in patients with alopecia. Br J Dermatol..

[3] Alfonso M, Richter-Appelt H, Tosti A, Viera MS, Garcia M. (2005). The psychosocial impact of hair loss among men: A multinational European study. Curr Med Res Opin..

[4] Williamson D, Gonzalez M, Finlay AY. (2001). The effect of hair loss on quality of life. J Eur Acad Dermatol Venereol..

[5] Reid EE, Haley AC, Borovicka JH, Rademaker A, West DP, Colavincenzo M (2012). Clinical severity does not reliably predict quality of life in women with alopecia areata, telogen effluvium, or androgenic alopecia. J Am Acad Dermatol..

[6] Finlay AY, Khan GK. (1994). Dermatology Life Quality Index (DLQI) - a simple practical measure for routine clinical use. Clin Exp Dermatol..

[7] Zhang M. (2017). Quality of life assessment in patients with alopecia areata and androgenetic alopecia in the People's Republic of China. Patient Prefer Adherence.

[8] Qi S, Xu F, Sheng Y, Yang Q. (2015). Assessing quality of life in alopecia areata patients in China. Psychol Health Med..

[9] Cartwright T, Endean N, Porter A. (2009). Illness perceptions, coping and quality of life in patients with alopecia. Br J Dermatol..

